# Vertical farming of hardy nursery stock: LED-driven propagation strategies compared with industry practice

**DOI:** 10.3389/fpls.2025.1697104

**Published:** 2026-01-20

**Authors:** Kambiz Baghalian, Ruvini Ranasingha

**Affiliations:** 1 Writtle School of Agriculture, Animal and Environmental Sciences, Faculty of Sciences and Engineering, Anglia Ruskin University, Chelmsford, United Kingdom; 2 School of Agriculture, Animal and Environmental Sciences, Anglia Ruskin University, Chelmsford, United Kingdom

**Keywords:** chlorophyll stability, controlled environment systems, hardy nursery stock, LED lighting effects, vertical farming, woody plant propagation

## Abstract

Hardy nursery stock production relies on the propagation of semi-hardwood or hardwood cuttings, yet rooting success is often inconsistent due to species-specific constraints and environmental variability. This study examined how vertical farming systems influences early propagation traits in three evergreen woody ornamentals—*Elaeagnus × ebbingei*, *Pittosporum tenuifolium*, and *Euonymus japonicus*—representing varying rooting difficulty. Cuttings were propagated under ambient light (control) or two light-emitting diode (LED) treatments differing in red-to-blue ratio and intensity (7:1 at 100 μmol m^-^² s^-^¹; 9:1 at 140 μmol m^-^² s^-^¹). Fresh shoot weight, fresh root weight, and chlorophyll content were measured, and trait stability assessed via coefficients of variation. *E. japonicus* achieved highest biomass under the 7:1 ratio, while *E. × ebbingei* and *P. tenuifolium* performed best under ambient light. Chlorophyll declined under LEDs in all species but remained most stable in *E. japonicus*. Variability analysis indicated chlorophyll as the most stable trait, shoot biomass moderately variable, and root biomass the most plastic. Principal component analysis indicated that chlorophyll and biomass traits were regulated independently, suggesting that rooting responses were more closely associated with carbon allocation processes than with pigment stability. These results demonstrate that species-specific responses shape propagation outcomes, and tailored LED strategies can enhance rooting uniformity and efficiency.

## 1 Introduction

In the ornamental nursery production sector, hardy nursery stock is a cornerstone of the trade, supplying landscape plants for public green spaces, private gardens, and commercial developments. This category includes outdoor-tolerant woody and semi-woody plants—such as shrubs, trees, and some perennials—that can survive year-round without protection in the prevailing climate (Hartmann et al., 2017). Their propagation generally relies on semi-hardwood or hardwood cuttings, with success depending heavily on efficient, high-quality techniques.

However, compared to less lignified cuttings such as softwood or herbaceous material, these more mature forms are inherently slower to root and present greater challenges during propagation. Rooting success is highly species-dependent, with some taxa responding relatively well while others are significantly more difficult to root. Moreover, these cuttings are more susceptible to seasonal limitations and heightened sensitivity to environmental stressors such as temperature and humidity fluctuations. Despite their commercial prevalence, such cutting-based methods remain constrained by biological and environmental variability, limiting reproducibility across propagation cycles (Chong, 1999; Hartmann et al., 2017). Moreover, fluctuations in environmental factors such as temperature and carbon dioxide levels have been shown to interact with hormone treatments, further affecting root development and the reproducibility of outcomes (Poudel and Dunn, 2018).

In addition to these environmental factors, several light-related factors play a decisive role in the rooting success of vegetative cuttings. Light quality (particularly the balance of red and blue wavelengths), light intensity, and photoperiod are all known to regulate physiological processes associated with early root initiation, including carbohydrate mobilisation, photoreceptor signalling, and auxin transport. These effects tend to be more pronounced in herbaceous and softwood material, where tissues are non-lignified, metabolically active, and more responsive to shifts in photosynthetic light cues. By contrast, semi-hardwood and hardwood cuttings—typical of hardy nursery stock—are partially lignified and adjust more slowly to spectral environments, making them more vulnerable to suboptimal light regimes during the first weeks of propagation. Understanding how light quality, intensity, and photoperiod interact with cutting type is therefore critical, and controlled LED-based systems offer a means of reducing the environmental noise that often undermines propagation uniformity.

The emergence of vertical farming systems, which enable precise control over environmental parameters, has created new opportunities to investigate spectral effects under reproducible and optimised conditions (Kaiser et al., 2024). Vertical farming systems have the potential to offer several production advantages that make them particularly suitable for propagation (Kaiser et al., 2024; Wai et al., 2024). By providing enclosed, multi-layered environments with uniformly controlled temperature, humidity, airflow, and lighting, these systems minimise the environmental fluctuations that often undermine rooting consistency in conventional glasshouse settings. Their high space-use efficiency and capacity for year-round production also present clear commercial benefits, particularly where predictable output and uniform plant quality are essential. Despite these advantages, vertical farming technologies still face limitations, including high energy demands, reliance on carefully optimised spectral strategies, and the need for species-specific refinement before widespread commercial adoption (Lorch-Schierning et al., 2024). Nonetheless, their capacity to deliver reproducible climatic and lighting conditions makes them a promising platform for advancing propagation research and improving the reliability of difficult-to-root nursery crops.

Among the components of vertical systems, lighting, has long been recognised as a critical factor in plant morphogenesis, influencing processes across developmental stages from seedling establishment to flowering and fruiting (Han et al., 2025).

Most existing studies on light spectra have focused on crop production, particularly examining later-stage traits such as biomass accumulation, flowering, and yield (Kaiser et al., 2024). Among these, research has been more common in herbaceous species (Kohler and Lopez, 2021), strawberries (Chen et al., 2025; Verma et al., 2024), and to some extent in ornamental plants (Dehestani-Ardakani et al., 2025; Gómez and Gómez, 2025). However, studies focusing on the propagation of woody cuttings under lighting recipes, or vertical systems remain especially scarce, with most existing studies focused on herbaceous or bedding plants propagation (Gómez and Gómez, 2025), highlighting the need for research in woody ornamental species.

In controlled environment agriculture, such spectral manipulations are most often implemented using LED fixtures, which allow fine control of wavelength composition, intensity, and photoperiod while maintaining high electrical efficiency. For example, Liu et al. (2023) showed that varying light durations under LED lighting significantly affected the growth of *Populus × euramericana* plantlets, illustrating how LED-based regimes can modulate development in woody material. Similarly, a recent study on *Lavandula angustifolia*, chosen as an initial model because it is a commercially relevant ornamental propagated from softwood cuttings, demonstrated that LED spectral composition influenced rooting efficiency and trait variability in vertical farming systems (Wai et al., 2024). Building on this foundation, the present work extends the investigation to semi-hardwood evergreen woody ornamentals with more challenging propagation requirements, thereby testing the robustness and broader applicability of vertical farming protocols across contrasting plant types.

Among various spectral components, red (R) and blue (B) wavelengths are particularly influential in modulating photosynthetic activity and morphological development by activating photoreceptors such as phytochromes and cryptochromes. These photoreceptors regulate key hormonal pathways, including auxin biosynthesis, transport, and localisation—processes fundamental to adventitious root initiation (Azizi et al., 2025). These effects are likely to be most influential during the early stages of cutting establishment, a period known to be particularly sensitive to light cues. For example, Guerrero-Sánchez et al. (2025) on *Ficus benjamina* demonstrated that exposure to R-B Light Emitting Diode (LED) spectra during the first few weeks of propagation significantly enhanced rooting traits, including root biomass and initiation rate. This suggests that the timing of light application, as well as spectral composition, is critical in modulating developmental outcomes, especially during the root induction phase. Similar timing strategies significantly enhanced vegetative growth in *Citrus*, with red–B LED exposure during winter also improving rooting (Bowman and Albrecht, 2021).

To assess the potential of vertical propagation systems equipped with LED lighting for woody ornamentals—particularly those classified as difficult-to-root—it is crucial to investigate species-specific trait responses. Understanding how different spectral environments influence traits such as rooting success, root biomass, and shoot development will clarify which conditions are most conducive to uniform propagation. Equally important is the evaluation of trait stability across lighting regimes, as consistency is a key determinant of commercial viability. High variability in outcomes not only undermines reproducibility but can also result in economic losses due to uneven plant quality or extended production cycles. By systematically comparing performance indicators under defined light treatments, it becomes possible to identify propagation environments that offer both high rooting efficiency and low trait variability—two key pillars of scalable commercial propagation.

This study investigated the early-stage responses of three commercially relevant evergreen woody ornamentals—*Elaeagnus × ebbingei*, *Pittosporum tenuifolium*, and *Euonymus japonicus*, to different lighting regimes, including ambient light and two LED lighting treatments. These species represent a gradient of propagation difficulty and commercial relevance. *Elaeagnus* is valued for its evergreen foliage, fragrant autumn flowers, and resilience to drought and pollution (Akoumianaki-Ioannidou et al., 2010). It is commonly propagated from semi-hardwood or hardwood cuttings depending on season, with rooting success influenced by genotype, timing, and growth regulator treatment (Sorokopudov et al., 2023). *Pittosporum* species, such as *P. tobira*, are widely used in landscape design for their compact form and drought tolerance but are generally more recalcitrant to rooting. Their propagation via hardwood or semi-hardwood cuttings often yields inconsistent results under conventional conditions, due to sensitivity to humidity, temperature, and light (Sayed et al., 2025). While *in vitro* auxin-based treatments have shown promise, commercial adoption is constrained by post-culture acclimatisation challenges (Maniati and Papafotiou, 2021). In contrast, *Euonymus japonicus* is regarded as relatively easy to root and is commonly used in hedging and landscaping (Sayed et al., 2025). These three species therefore provide a practical model to assess how light spectra influence adventitious root formation and associated physiological traits across differing levels of propagation difficulty.

These propagation challenges, particularly the inconsistency of rooting under uncontrolled environmental conditions, underscore the value of a more stable and controllable propagation environment (Lorch-Schierning et al., 2024; Wai et al., 2024). Vertical farming systems provide uniform lighting, temperature, humidity, and air movement, reducing environmental variability that commonly undermines rooting outcomes. As such, they offer a promising platform for testing species-specific spectral requirements and improving the reliability of woody cutting propagation (Kaiser et al., 2024).

By analysing shoot fresh weight (SFW), root fresh weight (RFW), and chlorophyll content (CC) under each lighting treatment, this study aims to (i) evaluate the species-specific effects of light quality on early growth and physiological development, (ii) quantify the degree of interaction between lighting and species on trait performance, and (iii) assess the consistency of these traits through coefficient of variation (CV%) analysis. This multifactorial approach is intended to inform species-tailored propagation strategies in controlled environments and contribute to the optimisation of vertical farming systems for woody plant production.

Overall, this study seeks to provide an evidence-informed basis for improving the reliability of woody cutting propagation under controlled environments and to support the gradual integration of vertical farming approaches into commercial nursery practice.

## 2 Material and methods

### 2.1 Plant material and experimental layout

Cuttings of three hardy nursery stock species—*Elaeagnus × ebbingei*, *Pittosporum tenuifolium*, and *Euonymus japonicus*—were obtained in collaboration with Palmstead Nurseries Ltd. (Ashford, Kent, UK; 51°10′53.41″ N, 0°55′05.17″ E) and selected for uniformity in size, physiological stage, and absence of visible stress or damage. The trial was conducted between October and December 2023 at the vertical farming research facility of Anglia Ruskin University (ARU), Writtle campus, Essex, UK (51°44′03.0″N, 0°25′50.1″E).

A total of 27 standard 60-cell propagation trays were used. Trays were filled with a propagation substrate supplied by Palmstead Nurseries, consisting of a fine-textured peat-based mix blended with a horticultural wetting agent and water-retention polymer to maintain moisture distribution and structural stability. The substrate contained a low nutrient charge to support rooting and establishment of woody evergreen cuttings. Each tray represented a biological replicate and therefore served as the experimental unit in the statistical analyses. Data from individual cuttings (ten per tray) were averaged to obtain a single value per trait per replicate. This approach avoids pseudo-replication and accounts for within-tray variability. Weekly tray rotation across shelves within each lighting compartment minimised positional effects associated with vertical light or airflow gradients, ensuring that each replicate experienced all shelf positions during the trial.

### 2.2 Lighting treatments

**Figure 1:**
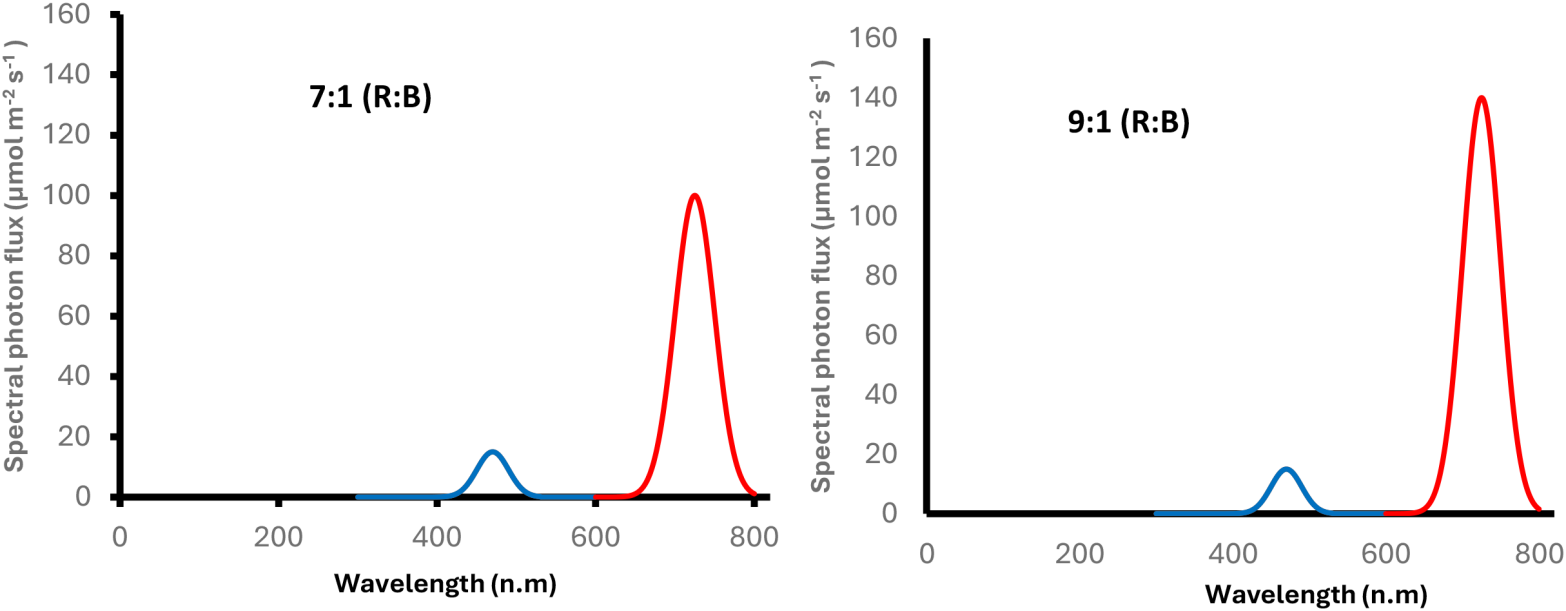
Spectral distribution of lighting for L1 (left) and L2 (right) treatments, illustrating the ratio of R:B.

The vertical farming chamber was fitted with two distinct LED lighting regimes, designated L1 and L2. A light-proof plastic partition ensured spectral isolation and treatment integrity. Their respective R:B ratios (7:1 and 9:1) and spectral profiles are shown in **Figure 1**. The selection of these two LED spectral ratios was based on existing literature and identified knowledge gaps, and further justification is provided in the Discussion section. Lighting conditions were monitored using a PAR meter, with Photosynthetic Photon Flux Density (PPFD) recorded at plant height via a computerised Sensor SpectroSense 2+ (Skye Instruments, UK).

Environmental setpoints were maintained uniformly across treatments, including day/night temperatures of 20/15°C, 75% relative humidity, and a fixed 16-hour photoperiod (**Table 1**). Within the vertical farming units, temperature and humidity were recorded hourly using TinyTag data loggers (Gemini Data Loggers, UK). Irrigation was provided by a closed ebb-and-flow system.

**Table 1:** Lighting regime and climate conditions used during trial.

Lighting treatment	Light ratio (R^a^ : B^b^)	LED light intensity (µmol m^-2^ s^-1^)^c^	Photoperiod (h^d^)	Temperature (°C)	
			(Night + Day)	Day	Night
L1	7:01	100	(8 + 16)	20	15
L2	9:01	140	(8 + 16)	20	15
Control (C)	Ambient light	n.a	Natural photoperiod	20	15

n.a., non applicable.

a Red.

b Blue.

c Micro moles per square meter per second.

d Hour.

Control groups for each species were placed on a glasshouse bench under ambient light for the same duration. During this phase, natural lighting and uniform environmental conditions were maintained. Temperature and humidity in the glasshouse were regulated and recorded using the Tomtech 200 automated system, replicating standard commercial practice aimed at producing cuttings of marketable size.

### 2.3 Data collection

Data were collected over the course of the trial from October to December 2023. Fresh root weight (FRW) and fresh shoot weight (FSW) were measured at three time points, spaced at biweekly intervals, to track rooting and shoot development. All destructive sampling and associated measurements were carried out within a consistent late-morning window on each sampling date to minimise diurnal variation in plant water status and fresh weight. At each of these sampling points, thirty cuttings per treatment were randomly selected—ten cuttings from each replicate tray. Cuttings that had initiated root development were gently extracted from the substrate, rinsed to remove residual growing media, and separated into root and shoot fractions. Fresh weights were recorded immediately using a precision balance.

Chlorophyll content (CC) was assessed non-destructively using a Konica Minolta SPAD-502 Plus chlorophyll meter at the start (D1) and end of the trial (D2). All SPAD measurements were also taken during the same late-morning time window to avoid diurnal fluctuations in chlorophyll index values. For each tray, ten Soil Plant Analysis Development (SPAD) readings were taken from randomly selected, visibly healthy leaves greater than 3 cm in length. Only one reading was recorded per leaf, and measurements were distributed randomly across trays to minimise sampling bias.

### 2.4 Data analysis

The experiment followed a randomised block design with three biological replicates per treatment, corresponding to the three propagation trays described in Section 2.1. Data collected as outlined in Section 2.3 were statistically analysed using IBM SPSS Statistics version 14 (IBM Corp., Armonk, NY, USA). Each tray was treated as the experimental unit, and the use of averaged values from ten randomly selected cuttings per tray prevented pseudo-replication while accounting for within-tray variability. Descriptive statistics (means and standard deviation) were calculated for all measured traits. Two-way analysis of variance (ANOVA) was conducted to evaluate the effects of lighting treatment, species, and their interaction on FRW, FSW, and CC. Where statistically significant differences were found (*P* < 0.05), Tukey’s Honestly Significant Difference (HSD) test was used for *post hoc* multiple comparisons.

Due to its physiological importance and potential sensitivity to spectral composition, CC (SPAD values) underwent additional statistical evaluation. Temporal stability was assessed using paired t-tests to compare SPAD values between D1 and D2 data collection points, both across all treatments combined and within each lighting treatment (C, L1, and L2) independently. In addition, species-level differences in baseline CC (D1) were assessed using one-way ANOVA, with species as the independent factor. Tukey’s HSD test was applied *post hoc* to identify statistically distinct species groupings, which were visualised as superscript letters in the chlorophyll bar chart.

In addition, Pearson correlation coefficients were calculated to assess relationships among measured traits. Principal Component Analysis (PCA) was used to explore multivariate trait variation and identify dominant axes of differentiation across treatments. Coefficients of variation (CV%) were also calculated to quantify relative variability within treatment groups.

## 3 Results

### 3.1 Differential effects of lighting and species on growth and chlorophyll traits

Two-way ANOVA revealed (**Table 2**) that both lighting (p < 0.05) and species (p < 0.001) had highly significant effects on SFW, with a strong interaction also observed between these factors (p < 0.001). RFW was significantly influenced by both lighting (p < 0.01) and species (p < 0.001), but their interaction was not statistically significant. Chlorophyll showed highly significant responses to all factors tested, including lighting, species, and their interaction (p < 0.001 for all).

**Table 2:** ANOVA significance levels for the effects of lighting, species, and their interaction on measured features.

Feature (measured)	Lighting	Species	Lighting × species
SFW	*	***	***
RFW	**	***	n.s.
CC	***	***	***

*p < 0.05; **p < 0.01; ***p < 0.001; n.s., not significant.

### 3.2 Species-specific responses to lighting treatments

**Figure 2:**
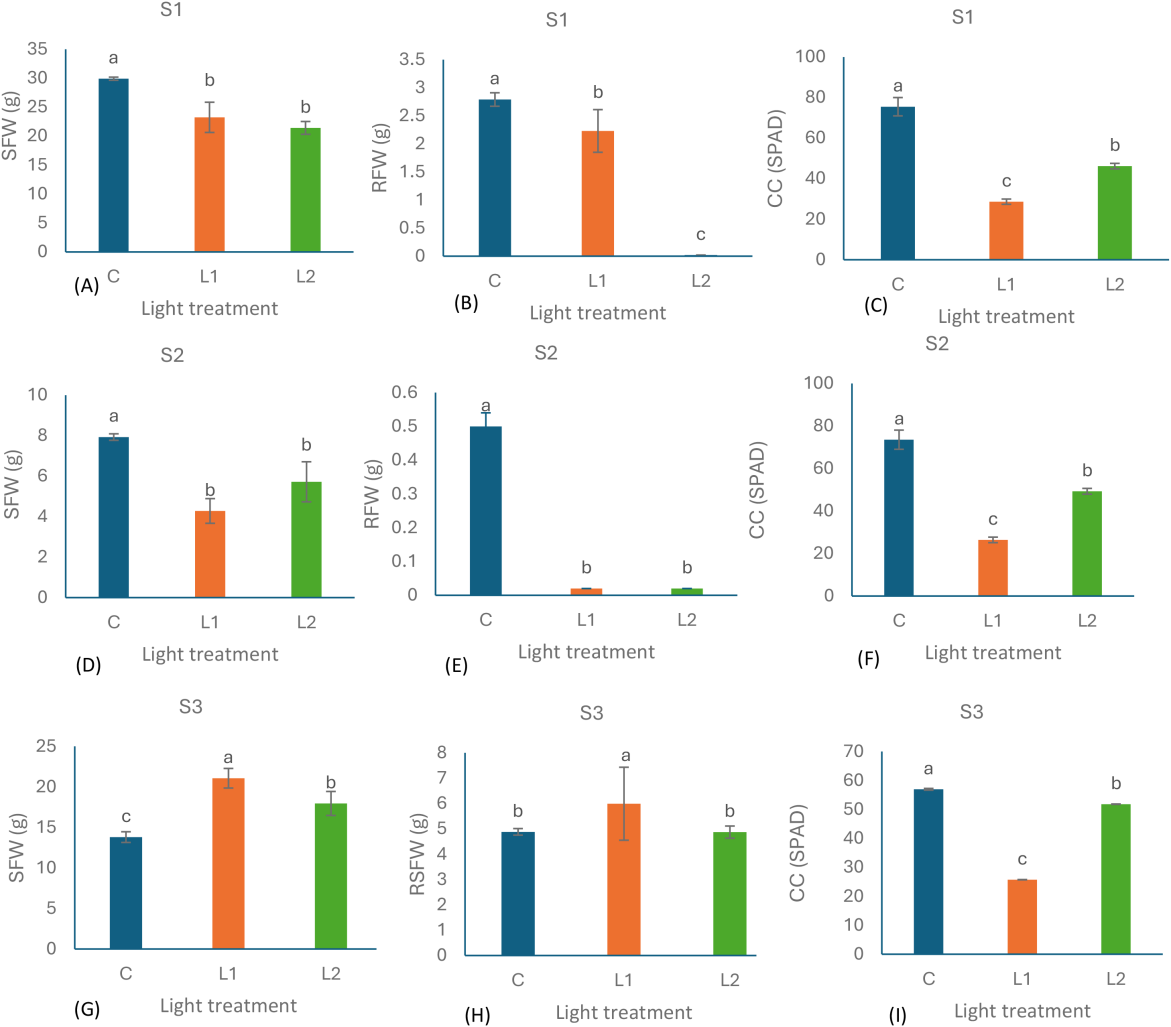
Effects of lighting treatments on shoot fresh weight (SFW), root fresh weight (RFW), and chlorophyll content (CC) across three woody ornamental species — *Elaeagnus × ebbingei* (S1; **A–C**), *Pittosporum tenuifolium* (S2; **D–F**), and *Euonymus japonicus* (S3; **G–I**) --under Control (C), L1 (7:1 at 100 µmol m^⁻²^ s^⁻¹^), and L2 (9:1 at 140 µmol m^⁻²^ s^⁻¹^) conditions. Bars represent mean ± standard deviation (n = 30 cuttings per treatment; ten cuttings per biological replicate). Different letters above bars indicate significant differences among lighting treatments within each species, determined by two-way ANOVA followed by Tukey's HSD test (p < 0.05).

**Figure 2** visualises the species-specific responses to lighting treatments for SFW, RFW, and CC, presenting the mean ± SD along with statistical groupings. These patterns highlight divergent trends across species under different lighting conditions.

Consistent with the significant interaction effect detected in the ANOVA for SFW and CC (p < 0.001 for both) (**Table 2**), the visual data in **Figure 2** illustrate clear species-specific responses to lighting treatments. Both S1 (**Figures 2A–C**) and S2 (**Figures 2D–F**) performed best under the Control condition across all measured traits, particularly CC, which declined sharply under L1 and L2 (**Figures 2C, F**). In contrast, S3 (**Figures 2G–I**) achieved its highest SFW and RFW under L1, suggesting a degree of physiological optimisation under modified lighting. Notably, the reduction in CC for S3 under L1 and L2 was less pronounced compared to the substantial declines observed in S1 and S2 (**Figure 2I**).

Although the interaction effect for RFW was not statistically significant, **Figure 2H** shows that S3 consistently outperformed the other two species in RFW, suggesting an inherently stronger rooting ability. Notably, under L1, S3 produced both the highest SFW (**Figure 2G**) and RFW (**Figure 2H**) among all species–lighting combinations.

**Figure 3:**
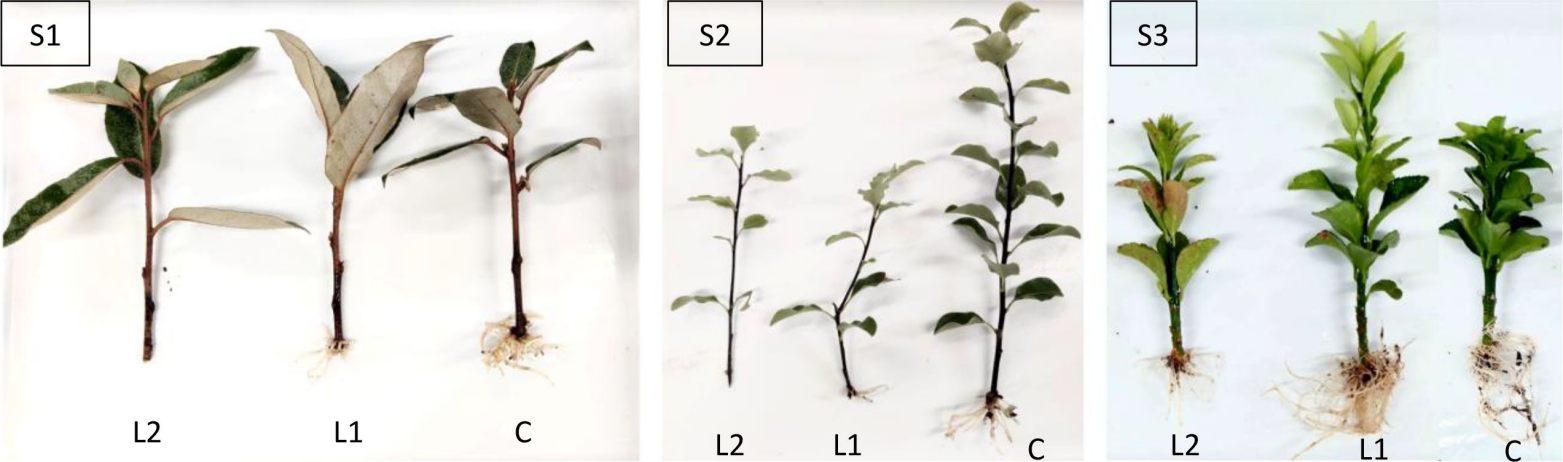
Root development of three woody ornamental species — *Elaeagnus × ebbingei* (S1), *Pittosporum tenuifolium* (S2), and *Euonymus japonicus* (S3) — under the three lighting treatments: Control (C), L1 (7:1 R:B), and L2 (9:1 R:B), photographed at the end of the trial.

This superior rooting performance is visually evident in **Figure 3**, which illustrates root development differences among the three species under varying lighting treatments.

#### 3.2.1 Lighting effects on chlorophyll accumulation

Although the ranking of CC across treatments was consistent in all three species (Control > L2 > L1), the magnitude of decline varied, indicating species-specific sensitivity to spectral composition, as illustrated in **Figures 2C, F, I**. S1 and S2 exhibited pronounced reductions under LED lighting, whereas S3 showed comparatively moderate declines, particularly under L2. As shown in **Table 2**, CC varied significantly across lighting treatments (p < 0.001). To further investigate these patterns, additional analyses was performed focusing on baseline species-specific differences independent of treatment or time effects (Section 1.3.1) and the temporal dynamics of CC (Section 1.3.2).

#### 3.2.2 Baseline differences in among species

**Figure 4:**
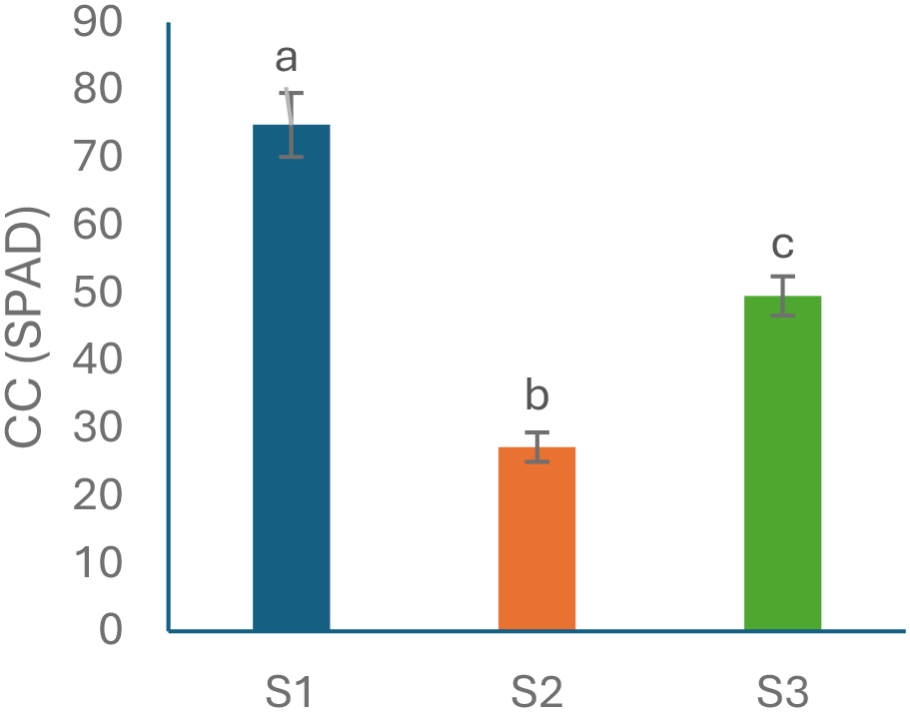
Baseline chlorophyll content (CC, expressed as SPAD index) for *Elaeagnus × ebbingei* (S1), *Pittosporum tenuifolium* (S2), and *Euonymus japonicus* (S3) measured at the first sampling point (D1), prior to treatment effects. Bars represent mean ± standard deviation (n = 30 cuttings per species; ten cuttings per tray, three biological replicates). Different letters above the bars indicate significant differences among species, determined by one-way ANOVA followed by Tukey’s HSD test (p < 0.05).

D1 was used as a baseline to capture inherent species-specific differences in CC, independent of treatment or time effects. By removing the influence of lighting treatments and temporal changes, this comparison reflects only species-specific variation. CC differed significantly among the three species (**Figure 4**) by S1 exhibited the highest mean CC (74.84 ± 4.73), followed by S3 with intermediate content (49.56 ± 2.90), while S2 showed the lowest content (27.22 ± 2.17).

To examine the temporal dynamics of CC more closely, data were first pooled across species, and a paired t-test indicated that the change in CC between the two sampling points (D1 and D2) was insignificant, suggesting stability over time.

### 3.3 CV% analysis of trait stability across treatments

To evaluate the relative stability of measured traits across lighting treatments and species, the CV% was calculated for SFW, RFW, and chlorophyll content.

**Figure 5:**
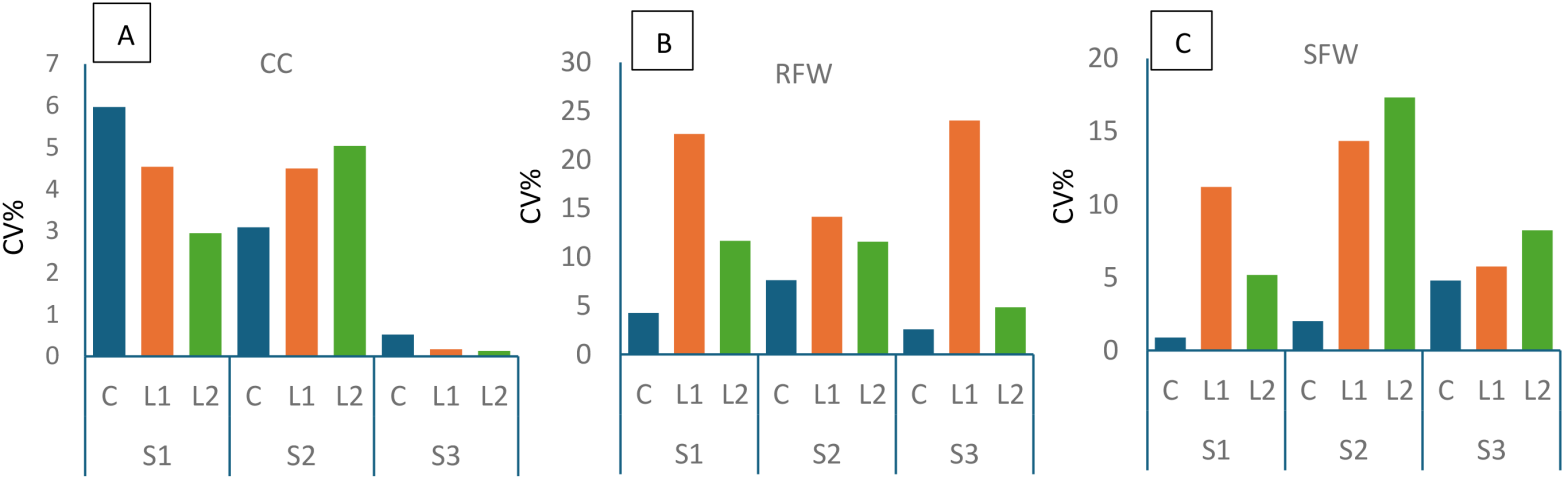
Coefficients of variation (CV%) for **(A)** chlorophyll content (CC), **(B)** root fresh weight (RFW), and **(C)** shoot fresh weight (SFW) across three lighting treatments — Control (C), L1 (7:1 R:B), and L2 (9:1 R:B) — in *Elaeagnus × ebbingei* (S1), *Pittosporum tenuifolium* (S2), and *Euonymus japonicus* (S3). Bars represent relative variability among three biological replicates (n = 30 cuttings per treatment).

As shown in **Figure 5A**, CC exhibited the lowest variability overall, particularly in species S3, where CV% remained below 1% across all lighting treatments.

In contrast, **Figure 5B** shows that RFW had the greatest variability, especially under L1 in S1 and S3 (CV = 22.65% and 24.03%, respectively).

**Figure 5C** illustrates moderate variation in SFW, with the highest CV% observed in S2 under L1 and L2 (17.33% and 16.27%, respectively), indicating greater sensitivity to LED lighting compared to the control (14.87%) for shoot development. Notably, C consistently yielded the lowest CV% for both SFW and RFW across all species (**Figures 5B, C**), reinforcing the role of artificial lighting as a source of physiological variation.

### 3.4 Trait relationships across treatments

Pearson correlation analysis (**Table 3**) revealed a moderate and statistically significant positive correlation between SFW and RFW (r = 0.42, *p* < 0.05). In contrast, no significant correlations were detected between CC and either biomass trait (SFW or RFW).

**Table 3:** Pearson correlation coefficients among traits.

	SFW	RFW	CC
SFW	1.00		
RFW	0.42*	1.00	-0.07
CC	0.11	-0.07	1.00

*p < 0.05.

**Figure 6:**
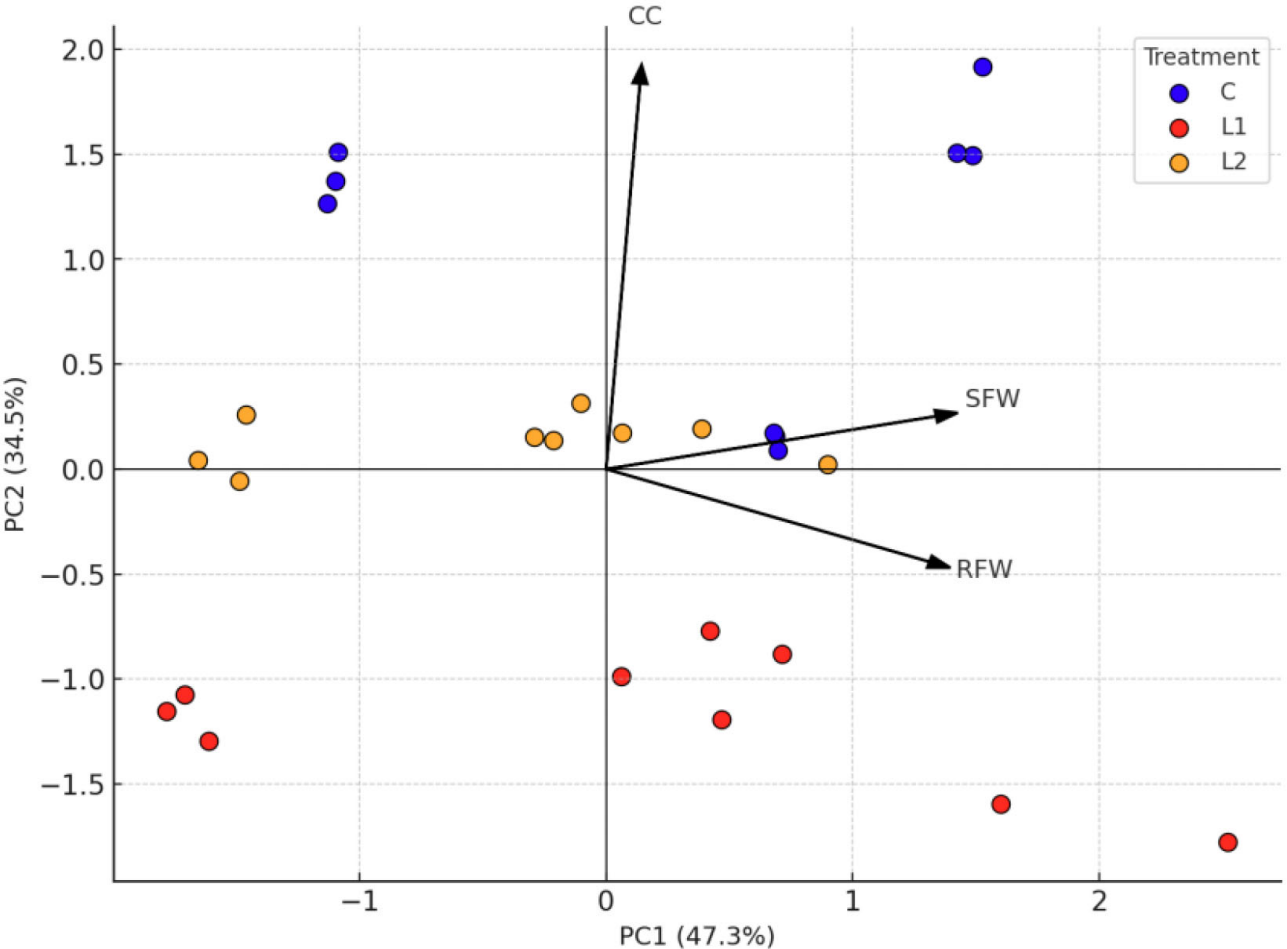
PCA biplot showing sample distribution across lighting treatments — Control (C), L1, and L2 — and trait vectors FSW, FRW, and CC.

To synthesise the overall structure of variation among traits across species and lighting treatments, a PCA was conducted. The first two principal components (PC1 and PC2) accounted for a combined 81.9% of the total variance, with PC1 explaining 47.5% and PC2 explaining 34.4%. SFW and RFW were the primary contributors to PC1, with loadings of 0.713 and 0.698, while CC dominated PC2 (loading 0.963), while CC dominated PC2 (loading: 0.963). (**Figure 6**) illustrates these relationships, showing that control samples clustered along the chlorophyll axis (PC2), whereas L1 and L2 treatments exhibited greater dispersion along the biomass axis (PC1), indicating distinct responses to lighting regimes.

## 4 Discussion

### 4.1 Species-specific responses and light interactions

Two-way ANOVA revealed significant effects of both lighting and species on all measured traits, with species consistently contributing the strongest main effects (**Table 2**). Visual trends shown in **Figure 2** reflect these interaction effects, highlighting how spectral treatments elicited distinct morphological and physiological responses depending on species. Together, these findings confirm that variation in response traits was structured by both species identity and lighting regime.

The two LED spectra were selected to represent physiologically meaningful and commercially relevant red–blue balances commonly applied in controlled-environment propagation. Blue wavelengths are associated with photomorphogenic signalling, chlorophyll synthesis, and early root initiation, whereas red wavelengths promote biomass accumulation and shoot elongation; such wavelength-dependent responses have been reported under red–blue LEDs in both herbaceous and woody species, including evidence for photoreceptor activation, chlorophyll enhancement, and early developmental regulation (Azizi et al., 2025; Djangalina et al., 2023). Although numerous studies have examined red–blue light responses in herbaceous crops and bedding-plant cuttings, substantially fewer investigations have focused on woody ornamental species, leaving uncertainty regarding their early physiological behaviour under contrasting spectral environments. Recent findings from *Euphorbia leucocephala* stem-cutting propagation demonstrate that spectral composition can significantly influence adventitious root formation and early shoot development (Morales-Becerril et al., 2025), further emphasising the need for broader evaluation across additional woody taxa. In light of this sparse evidence base and the recognised knowledge gap for woody material, two contrasting red–blue spectral ratios were selected to determine whether differences in spectral balance influence early physiological responses across species differing in propagation difficulty.

These results align with previous studies demonstrating that plant responses to LED treatments are mediated by interactions between spectral composition and intrinsic physiological traits such as photoreceptor sensitivity, pigment biosynthesis, and hormonal dynamics (Azizi et al., 2025). In *Citrus* cultivars, for example, R–B LED combinations enhanced growth in some genotypes but not others, underlining the influence of genotype-specific light responsiveness (Bowman and Albrecht, 2021).

Species with lower baseline chlorophyll levels at the start of the trial (**Figure 4**), such as S2, may have inherently reduced chlorophyll concentrations prior to any spectral exposure. Such species may be more sensitive to R-dominant light, potentially due to physiological traits that affect chlorophyll stability or hormonal responsiveness. This interpretation is consistent with trait stability patterns (**Figure 5A**) and the separation of samples along the chlorophyll axis in the PCA biplot (**Figure 6**), as well as the absence of significant correlations between CC and fresh weight traits (**Table 3**). These findings align with the work of (Guerrero-Sánchez et al.), who demonstrated species-specific differences in rooting efficiency and auxin levels under varying light spectra in *Pittosporum tobira*, *Photinia fraseri*, and *Camellia japonica*.

These patterns contrast with responses observed in softer vegetative cuttings such as strawberry runners, where LED-enriched spectra improved both shoot biomass and CC across tipping dates (Wai et al., 2024). This divergence likely reflects differences in cutting physiology, as strawberry runners, being non-lignified and reliant on high metabolic turnover, respond more directly to photosynthetic enhancement, whereas semi-hardwood cuttings of the current trial showed more constrained chlorophyll responses under spectral stress.

Additional insight comes from Populus (Mogilevskaya et al., 2025), where varying R:B ratios and intensities produced divergent outcomes across clones for traits such as shoot biomass, stem elongation, and leaf expansion. Collectively, these results reinforce that LED spectral responses are not uniform across species or traits, and that optimisation of both light quality and intensity must be tailored to the physiological architecture of each species or cultivar.

These species-level physiological insights provide a foundation for exploring how CC varied over time and under specific spectral conditions, as described in Section 4.2.

### 4.2 Chlorophyll stability under spectral variation

To assess temporal chlorophyll dynamics, CC was monitored over time in response to different light intensities and spectra. When data were pooled across treatments, no significant difference was observed between D1 and D2, confirming that chlorophyll accumulation remained temporally stable under trial conditions. However, a significant decline under L2 (p < 0.05), but not under L1 or control conditions, suggests that R-enriched, high-intensity light exerts a greater depleting effect on chlorophyll pools (Section 1.3.2).

In contrast, lavender tip cuttings propagated under comparable R:B LED ratios maintained both chlorophyll stability and improved rooting (Wai et al., 2024). This discrepancy may be attributed to cutting type, as leafy tip cuttings retain higher photosynthetic activity and carbohydrate export during rooting, whereas semi-hardwood cuttings, despite leaf retention, may exhibit reduced assimilation capacity due to partial lignification and limited stomatal conductance.

These patterns reinforce baseline species differences (**Figure 4**). For example, S3, which started with intermediate chlorophyll levels, maintained chlorophyll stability under L1 while achieving notable fresh-weight gains (**Figures 2G, H**). This suggests that moderate baseline chlorophyll may buffer chlorophyll loss under milder spectral conditions. Although chlorophyll content and fresh-weight traits were regulated independently (see Section 4.4), the co-occurrence of chlorophyll depletion and root variability under certain treatments (**Figure 5B**) suggests physiological crosstalk—possibly via hormonal or photosynthetic pathways.

Comparable findings were observed in Vitis vinifera, where white LEDs supported chlorophyll stability while R-rich treatments promoted growth despite lowering chlorophyll levels (Coşar Tutumlu and Topcu Altıncı, 2025). Together, these data indicate that pigment content is relatively stable temporally but susceptible to intensity-related stress and may serve as a useful early diagnostic marker.

While the experiment maintained comparable temperature and humidity setpoints across all treatments, it is acknowledged that the physical environments of the LED chambers and the glasshouse control were not identical. The enclosed vertical system operated with limited air exchange, whereas the glasshouse allowed greater natural airflow and fluctuating CO_2_ levels. Such differences could have influenced microclimatic factors such as boundary-layer conductance, vapour pressure deficit, and CO_2_ diffusion near the leaf surface, which in turn may have affected photosynthetic rate and pigment stability. Although these potential discrepancies were partially mitigated through uniform temperature–humidity control and tray rotation, they remain a limitation of the present study. Future work employing enclosed control compartments or controlled CO_2_ supplementation would help isolate the specific effects of light spectra from background environmental variation. The relationship between chlorophyll stability and trait variability is further evaluated in Section 4.3, which shifts focus to the differential plasticity of root and shoot traits.

### 4.3 Biomass plasticity and rooting sensitivity

Root and shoot traits displayed markedly higher plasticity under spectral treatments compared to CC, as quantified by CV% (**Figure 5**). While CC remained stable across lighting treatments, particularly in S3 (CV% <1%), RFW showed substantial variability, peaking at 22.7–24.0% in S1 and S3 under L1. This indicates that morpho-physiological traits differ in responsiveness to spectral cues.

Studies in lavender and strawberry support this pattern, in which non-lignified tip cuttings exhibited strong rooting responses under LED lighting while maintaining stable chlorophyll levels (Lorch-Schierning et al., 2024; Wai et al., 2024). Their high metabolic activity and photosynthetic reliance contrast with the slower, reserve-driven rooting of semi-hardwood cuttings, such as those examined here.

Evidence from *Euonymus* and *Elaeagnus* further highlights broad genotypic variation in rooting capacity even under standardised hormone regimes (Poudel and Dunn, 2018; Sorokopudov et al., 2023). Similar patterns emerged here, with S1 exhibiting high root variability (**Figure 5B**). Increased CV% under LED treatments relative to controls suggests that artificial lighting amplifies physiological noise, potentially complicating uniform propagation.

SFW showed intermediate plasticity (17.3% CV in S2 under L2), consistent with findings in *Sorbus domestica* and *Malus domestica* where spectral treatments induced greater variability in rooting and elongation compared to stable pigment indices (Lupo et al., 2025; Tomazini Scolaro et al., 2025). Collectively, these findings establish a gradient in which chlorophyll remains stable, SFW is moderately variable, and root traits are the most plastic under spectral manipulation.

This gradient leads directly to Section 4.4, which quantitatively dissects trait independence using PCA and correlation analyses.

### 4.4 Independent regulation of fresh-weight traits and chlorophyll content

Building on the CV% findings in section 4.3, which highlighted the contrasting stability of CC and the greater plasticity of fresh-weight traits (**Figure 5**), correlation and PCA provided further evidence that these traits are regulated independently. Pearson correlation analysis (**Table 3**) revealed a significant positive association between SFW and RFW (r = 0.42, p < 0.05), whereas CC showed no significant correlation with either fresh-weight trait, underscoring the decoupling of chlorophyll stability from growth plasticity. PCA supported this independence, with shoot and root fresh weight loading strongly on PC1 (0.713 and 0.698, respectively), which explained 47.5% of the variance, while CC loaded exclusively on PC2 (0.963), which accounted for 34.4% of the variance (**Figure 6**).

Evidence from other propagation contexts reinforces this distinction. In lavender, rooting gains under LED lighting occurred alongside stable chlorophyll levels (Wai et al., 2024), whereas in strawberry runners, biomass accumulation and pigment retention were more closely aligned (Lorch-Schierning et al., 2024), likely reflecting their greater reliance on immediate photosynthetic activity during early rooting. While neither study employed PCA, these contrasting patterns may support the interpretation that cutting type modulates how chlorophyll stability and biomass traits interact under spectral manipulation. Leafy, non-lignified cuttings respond more synchronously to chlorophyll -driven photosynthetic cues, whereas semi-hardwood cuttings appear to rely more on stored reserves during early rooting.

Control samples clustered predominantly along the chlorophyll axis (PC2), while LED-treated samples (L1 and L2) dispersed along the fresh-weight axis (PC1), indicating divergent responses of chlorophyll and growth traits under spectral manipulation (**Figure 6**).This independence helps explain why S3, despite its intermediate baseline chlorophyll levels (49.56 ± 2.90; **Figure 4**), displayed superior rooting performance under L1 (**Figures 2H**, **3**). Its rooting gains likely reflect physiological mechanisms distinct from chlorophyll accumulation, consistent with its low chlorophyll variability under LED treatments (CV% <1%; **Figure 5A**). Comparable decoupling has been documented in *Vitis vinifera*, where biomass gains and chlorophyll retention were uncorrelated under varying light spectra (Coşar Tutumlu and Topcu Altıncı, 2025). Similarly, *Sorbus domestica* exhibited enhanced rooting and elongation under R–B LEDs without significant shifts in pigment indices (Lupo et al., 2025), while in *Cunninghamia lanceolata*, PCA revealed separate clustering of pigment and root traits under contrasting light spectra *(*Xu et al., 2020*)*.

Together, these findings confirm that chlorophyll stability and fresh-weight development are regulated through distinct pathways that respond differently to spectral cues—supporting the use of PCA and trait-specific variability analyses as tools to optimise propagation strategies.

### 4.5 Light-mediated physiological mechanisms in rooting

The marked variability in RFW under LED treatments—particularly in S1 and S3—combined with the PCA separation of rooting from chlorophyll traits (**Figure 6**), indicates that root development was regulated largely independently of chlorophyll stability. As shown in **Figure 5**, RFW displayed the greatest plasticity, especially in S1 and S3 under L1, whereas S2 exhibited more constrained rooting responses despite comparable shoot fresh weight. This species divergence suggests that rooting responses were primarily governed by physiological processes linked to photosynthetic supply and hormonal signalling rather than pigment accumulation.

Rooting success in woody cuttings has been closely associated with the functional role of leaves, which serve both as sites for auxin absorption and transport and as sources of photosynthetic assimilates (Hartmann et al., 2017). In *Elaeagnus*, cuttings retaining more leaves showed enhanced photosynthetic activity and superior rooting performance (Sorokopudov et al., 2023), while in *Euonymus*, foliar auxin application improved rooting more effectively than basal dips (Poudel and Dunn, 2018).

Comparatively, strawberry runners rooted vigorously under LED lighting without significant pigment depletion (Wai et al., 2024), reflecting a physiology dominated by soft tissues and high auxin mobility. Similarly, lavender tip cuttings benefited from LED regimes that simultaneously promoted chlorophyll retention and rooting (Lorch-Schierning et al., 2024). These contrasts highlight how soft, leafy cuttings integrate spectral signals through photosynthetic and hormonal pathways more readily than semi-hardwood cuttings, where rooting plasticity is shaped more by stored carbohydrate reserves and slower hormonal transport.

These findings align with these trial’s results, where S1 and S3, which maintained low chlorophyll variability under LEDs, likely benefited from stable photosynthetic function supporting carbohydrate flow and auxin transport, thereby enabling greater rooting plasticity. In contrast, S2’s restricted rooting variability under LEDs may reflect limitations in integrating these pathways under spectral stress.

However, the present study did not include photosynthetic measurements such as gas-exchange or chlorophyll fluorescence, which would allow more direct validation of these physiological interpretations. Incorporating such measurements will form an important component of future investigations to strengthen the mechanistic linkage between spectral cues, photosynthetic performance, and rooting behaviour.

Light spectra further modulate rooting through hormonal regulation. Guerrero-Sánchez et al. (2025) demonstrated that R–B LEDs enhanced rooting in *Pittosporum* and *Photinia* via increased auxin-related activity, particularly during early propagation phases. Similarly, in *Sorbus domestica*, R-rich lighting promoted lateral root elongation through heightened auxin sensitivity (Lupo et al., 2025). Although *Pittosporum* responded well in these controlled studies, its weaker rooting response in current experiment may indicate genotype-specific spectral thresholds or differences in photoperiodic sensitivity.

Spectral quality also influences the auxin–cytokinin balance that drives root initiation. In *Malus domestica*, B light (450 nm) increased the auxin-to-cytokinin ratio, improving rooting, whereas R light (660 nm) was less effective (Tomazini Scolaro et al., 2025). This aligns with current trial’s findings for S3, where stronger rooting occurred under L1, which provided a more balanced B component compared to the R-enriched, higher-intensity L2 (**Table 1**, **Figure 1**).

Light intensity itself plays a decisive role in rooting sensitivity. In *Cunninghamia lanceolata*, moderate R–B intensities enhanced rooting, whereas higher intensities suppressed elongation and altered callus formation (Xu et al., 2019). Comparable patterns emerged in this study, where S1 and S3 achieved stronger rooting under L1 but showed increased variability and signs of suppression under the more intense, R-enriched L2 (**Table 1**; **Figure 1**; **Figure 5B**). Similarly, in *Eucalyptus*, Schilisting et al. (2025) found that light-induced callus formation did not consistently translate into rooting, highlighting that callogenesis and rhizogenesis follow distinct photomorphogenic pathways influenced by spectral cues.

Collectively, these findings indicate that the rooting plasticity observed in this study (**Figure 5**) reflects species-specific integration of spectral cues with photosynthetic and hormonal regulation, rather than being directly tied to chlorophyll stability. The PCA separation between chlorophyll and rooting traits (**Figure 6**) underscores this independence, emphasising that rooting performance under LED lighting is likely influenced by processes related to carbohydrate allocation and auxin dynamics. Therefore, effective propagation strategies should prioritise spectral conditions that stimulate these mechanisms, moving beyond chlorophyll -based indices as predictors of rooting success.

### 4.6 Practical implications for propagation protocols

The findings of this study provide actionable insights for refining propagation strategies under controlled lighting conditions. The marked interspecific differences in rooting responses (**Figures 2**, **3**; **Table 2**), coupled with the low correlation between CC and rooting traits (**Table 3**; **Figure 6**), highlight that propagation protocols should be customised according to species physiology and cutting type rather than adopting uniform spectral settings.

For leafy tip cuttings such as lavender, previous work has demonstrated that optimised R:B LED ratios (7:1 and 9:1) not only enhanced root emergence but also stabilised CC, with effects persisting post-potting (Wai et al., 2024). This supports using moderate R-enriched spectra during early propagation to balance rooting efficiency with chlorophyll retention in species where leaf photosynthesis is central to carbohydrate supply. Conversely, semi-hardwood cuttings, as represented by S1 and S2, exhibited greater rooting variability (**Figure 5B**) and chlorophyll depletion (**Figures 2C–F**), suggesting the need for milder spectral intensities or hybrid regimes combining natural and artificial light to reduce stress.

The propagation timing effects reported in strawberry further indicate that spectral optimisation must align with seasonal and physiological windows to ensure consistent rooting (Lorch-Schierning et al., 2024). Integrating chlorophyll monitoring (**Figures 2C–F**; **5A**) as a real-time diagnostic tool could help identify early stress signals, allowing for dynamic spectral adjustments before rooting variability escalates.

Finally, the evidence that early spectral conditioning enhances both rooting and subsequent transplant vigour (**Figures 2G–I**, **3**), supported by lavender’s improved post-potting performance (Wai et al., 2024), suggests that propagation lighting choices can have lasting commercial benefits. By combining trait-based diagnostics, cutting-type considerations, and targeted spectral regimes, these findings offer a framework for developing propagation protocols that improve rooting uniformity while enhancing downstream plant quality in nursery and vertical farming systems.

## 5 Conclusion

This study shows that LED lighting in vertical farming systems significantly influences propagation outcomes in woody ornamentals, with responses strongly dependent on species. While *Euonymus japonicus* achieved optimal fresh-weight gains under a 7:1 red-to-blue LED ratio, *Elaeagnus × ebbingei* and *Pittosporum tenuifolium* performed better under ambient conditions. Chlorophyll content proved to be the most stable trait, whereas RFW was highly variable, supporting the inference that rooting performance is more closely associated with carbon allocation and hormonal regulation than with pigment stability. Future work incorporating photosynthetic and fluorescence measurements will enable more robust mechanistic evaluation of how spectral environments influence early-stage physiological responses in woody cuttings.

From a horticultural perspective, these findings underline the potential of tailored spectral strategies to enhance the predictability and uniformity of nursery propagation. Improved consistency in rooting directly translates into reduced production losses, shorter propagation cycles, and higher market quality, offering clear commercial benefits for growers. Future work should refine spectral timing and intensity to target the root induction phase and extend trials across a broader range of nursery species. By integrating physiological insights with practical propagation protocols, this research contributes to the development of precision lighting strategies that strengthen the efficiency and sustainability of hardy nursery stock production.

## Data Availability

The raw data supporting the conclusions of this article will be made available by the authors, without undue reservation.
